# Evaluation of Implant Success in Patients with Dental Aplasia

**DOI:** 10.1155/2019/1680158

**Published:** 2019-06-19

**Authors:** Sameh Attia, Ella Schaper, Heidrun Schaaf, Jörn Pons-Kühnemann, Maximiliane Amelie Schlenz, Philipp Streckbein, Sebastian Böttger, Hans-Peter Howaldt, Jan-Falco Wilbrand

**Affiliations:** ^1^Department of Cranio-Maxillofacial Surgery, Faculty of Medicine, Justus-Liebig University Giessen, Klinik Str. 33, 35392 Giessen, Germany; ^2^Institute for Medical Informatics and Medical Statistics, Faculty of Medicine, Justus-Liebig University Giessen, Rudolf-Buchheim Str. 6, 35392 Giessen, Germany; ^3^Justus-Liebig-University Giessen, Department of Prosthodontics, Schlangenzahl 14, 35392 Giessen, Germany

## Abstract

**Introduction:**

Dental aplasia is an anomaly in which the number of teeth is reduced. It is the most commonly occurring dental anomaly during tooth development. Treatment management of patients with dental aplasia is challenging.

**Objectives:**

The aim of this retrospective clinical study was to analyze the survival and success rates of dental implants placed in hypodontic patients, rated with different criteria.

**Methods:**

Forty-three patients were diagnosed with dental aplasia and treated with dental implants between November 2000 and February 2016. The variables assessed included the plaque level, bleeding on probing, probing depth, implant mobility, implant stability, and implant loss. To analyze the peri-implant bone level, a panoramic X-ray of each patient was taken. The results were compared with X-rays taken immediately after implantation.

**Results:**

Thirty-seven patients (16 males; 21 females) participated in this study. In total, 155 implants (86 maxillary; 69 mandibular) were inserted. Two of the 155 implants failed; the* in situ* survival rate was 98.7%. The success rate according to the criteria of Buser et al. was 96.8%, and that according to the criteria of Albrektsson et al. was 88.4%.

**Conclusion:**

The survival and success rates of dental implants in patients with congenitally absent teeth were very high and did not differ significantly from results achieved in an unaffected population. Dental implants are a reliable therapy for patients with dental aplasia.

## 1. Introduction

Types of dental aplasia include hypodontia, oligodontia, and anodontia. Hypodontia is the absence of one to five teeth, and oligodontia is the absence of more than five teeth, excluding the wisdom teeth [1]. Anodontia is characterized by the partial or complete absence of deciduous and permanent dentition [2]. The prevalence of dental aplasia in the deciduous dentition varies among countries, with a reported range of 0.2% to 0.9% [3, 4]. Agenesis of the primary dentition is associated with an increased risk of tooth absence in the secondary dentition [5]. The prevalence of hypodontia in the permanent dentition is 2–10% [6, 7]. Several studies have documented an uneven sex distribution for dental aplasia, with a greater prevalence among females than among males [8–10]. The most frequently absent teeth in the permanent dentition are the mandibular second premolars (1–5%), maxillary lateral incisors (0.5–3%), maxillary second premolars (1–2.5%), and mandibular lateral incisors (0.5%). The prevalence of wisdom tooth absence is 10–35% [6]. Dental aplasia is associated with several syndromes, such as ectodermal dysplasia, cleft lip, cleft palate, Rieger syndrome, and Down's syndrome [11]. The etiology of hypodontia may involve genetic (nonsyndromal) factors [12]. Seven genes are known to be associated with the development of dental aplasia:* MSX1*,* PAX9*,* AXIN2*,* EDA*,* EDARADD*,* NEMO*, and* KRT17141* [13]. However, the exact etiopathogenesis of dental aplasia is not completely clear [14]. Although there is no clear relationship between dental aplasia and bone metabolic disease recorded, many clinical signs are generally observed. These clinical features include tooth morphology (microdontia) and tooth malposition in different manner such as infraocclusion of primary molars, ectopia, and transposition of permanent teeth [15, 16]. Dental aplasia can seriously affect young patients physically and psychologically, particularly during puberty. Interdisciplinary cooperation among dental practitioners is important to achieve optimal treatment outcomes for these patients [2]. Therapeutic options for dental aplasia depend on the number and location of absent teeth, dental implants, resin-bonded or conventionally fixed dental prostheses, autotransplantation sites, and sites of orthodontic tooth gap closure [14, 17–19]. Dental implant placement is a reliable and effective method for the rehabilitation of even augmented jaws [20]. A deciduous tooth can serve as a space maintainer until cranial bone growth is complete and a dental implant can be inserted [21].

Many studies have evaluated dental implants in patients with dental aplasia, with a focus on the implant survival rate [22–27]. Soft-tissue parameters were not evaluated in most of these studies, and implant success was evaluated with self-defined parameters. Standard success criteria were not used in any of these studies. The aim of the present retrospective study was to determine the success and survival rates of dental implants in patients with dental aplasia. Implant success was evaluated using the Buser and Albrektsson criteria [28, 29]. An individual questionnaire was used to collect general patient data and record patient satisfaction.

## 2. Materials and Methods

Forty-three patients with dental aplasia were treated with endosseous implants at the Department of Oral and Maxillofacial Surgery, University Hospital Giessen, Germany, during 2000–2016. Data collected from patient records included age, sex, number and location of absent teeth, and implant- and prosthetic-based rehabilitation. All selected patients who fulfilled the inclusion criteria were invited to undergo clinical and radiological examinations and an interview that included the administration of a customized questionnaire. The main purpose of the assessment was to evaluate implant success according to the criteria of Albrektsson and Buser [29, 30]. Implant success was defined as the fulfillment of all criteria, and implant failure was defined as the failure to satisfy at least one criterion. Explanted implants, regardless of the reason for removal, were also considered to have failed.

The following clinical parameters were recorded in the assessment of dental implant success: the modified Mombelli plaque index [31], probing depth measured using the Click-Probe (Kerr Corporation, Orange, CA, USA) [32], bleeding on probing (*the mobility grade inferred the osteointegration and stability and was calculated for each dental implant* using the Periotest® device (Gulden, Modautal, Germany)), and the absence or presence of keratinized gingiva (Table 1).

The presence of peri-implant infection was assessed clinically and radiologically at the same time and defined as the presence of a pocket depth ≥ 4 mm, bleeding on probing, and/or exudate and vertical bone loss > 1.5 mm + [0.2 mm × (years − 1)].

Vertical bone loss was determined by calculating the difference in alveolar bone height between panoramic X-rays taken immediately after implantation and at the follow-up examination. The presence of radiolucency around dental implants was assessed on the panoramic X-rays. To exclude measurement error, all panoramic X-rays were obtained with the same device (Sirona®, Bensheim, Germany) and evaluated by the same examiner. The data were collected from the patients' digital files (KAOS software, University Hospital Giessen clinical administration system) and categorized using Microsoft Excel® software (version 2017; Microsoft Corporation, Redmond, WA, USA).

The research ethics committee of the Faculty of Medicine, Justus Liebig University Giessen, approved this study (no. 209/15).

### 2.1. Statistical Analysis

Implant survival probability was calculated in a Kaplan–Meier analysis performed in collaboration with the Institute of Medical Informatics of Justus Liebig University Giessen using SPSS software (version 24.0; IBM Corporation, Armonk, NY, USA).* The Chi-square test (χ*^2^*)- or Fischer's more accurate test for categorical variables was applied to investigate the correlation between implant systems used, the type of graft, and age or sex of patients.*

## 3. Results

**Figure 1:**
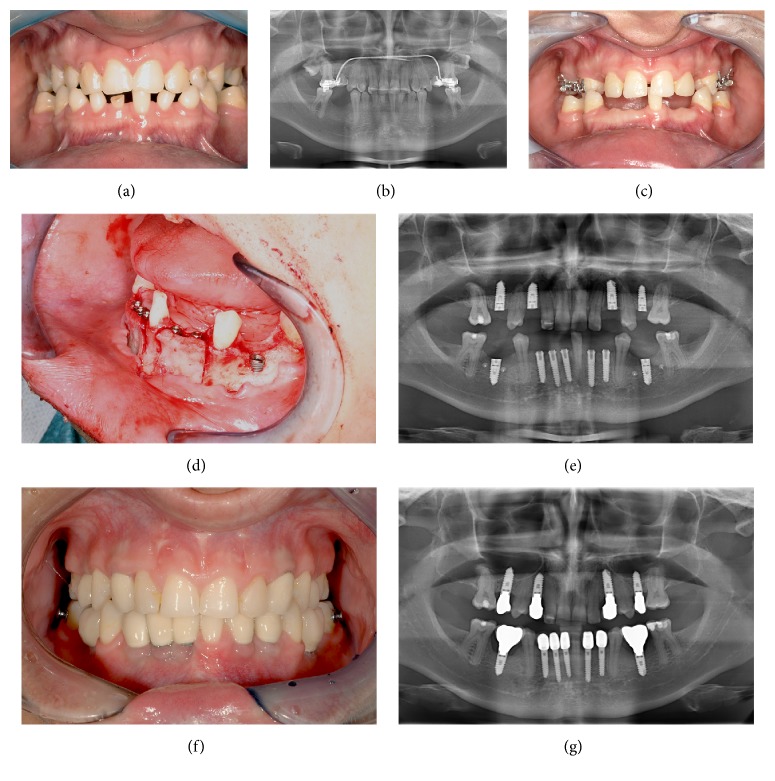
Treatment procedures of patient with oligodontia until oral rehabilitation. (a) Initial condition, patient with oligodontia (13 teeth absent). (b) Panoramic X-ray of the initial case. (c) After deciduous tooth extraction. (d) Intraoperative surgical view, insertion of implant and bone splitting. (e) Postoperative panoramic X-ray showing implant position. (f) Prosthetic rehabilitation with single tooth crowns. (g) Panoramic X-ray, 3 years after implantation.

Forty-three patients with hypodontia or oligodontia (25 females; 18 males) received dental implants for functional and aesthetic rehabilitation during November 2000–September 2016. All patients were treated surgically at the Department of Oral and Maxillofacial Surgery and prosthodontically at the Department of Prosthodontics of the University Hospital Giessen, Germany. Data on the patients' general condition and personal habits were collected at the time of the follow-up examination. Treatment outcomes were evaluated in 37 patients using a customized questionnaire during the clinical examination. Six patients refused to participate in the study and were counted as dropouts. Patient age at the time of implantation ranged from 17 to 44 years (mean, 21.4 years). The majority (*n* = 33) of patients treated with dental implants were young. Bone augmentation from the mandibular angle was performed in five (13.5%) patients with eight (5.2%) implants. Iliac crest bone grafts were used in 13 (35.1%) patients with 89 (57.4%) implants.  Figure 1 shows a patient with oligodontia and treatment procedures until oral rehabilitation.

### 3.1. Dental Implants and Survival

**Figure 2:**
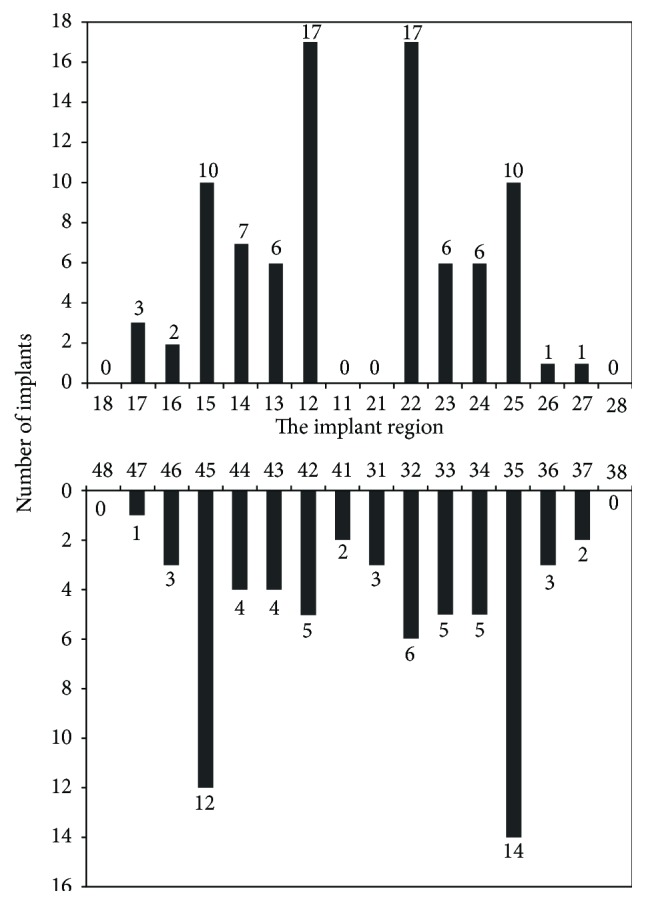
Implant distribution according to dental region.

In total, 155 implants (86 maxillary; 69 mandibular) were inserted (94 in males; 61 in females; Figure 2).

**Figure 3:**
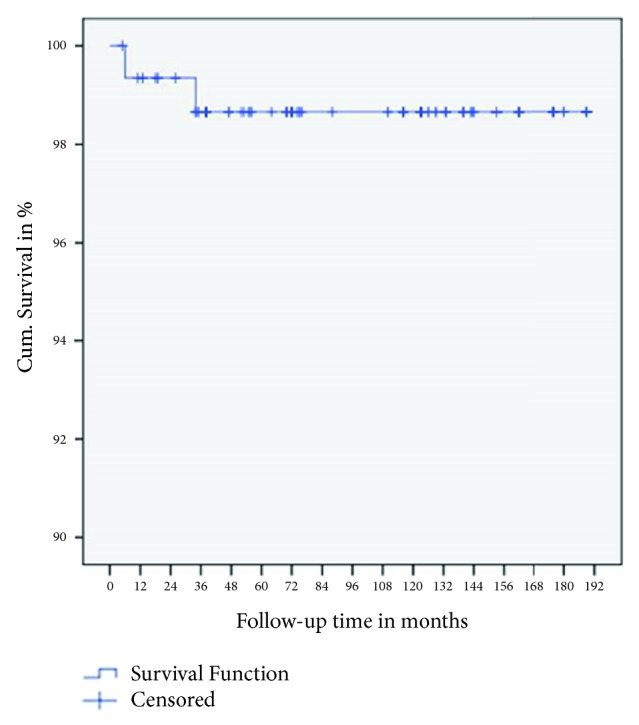
Cumulative Kaplan–Meier survival curve for dental implants.

Three different implant systems were used over the time of observation. In the beginning (Year 2000) mainly Straumann Standard® Implants with a parallel macrogeometry (Straumann, Basel, Switzerland; *n* = 10) were inserted. Within the following years (until 2016) mainly two different implant systems (Xive Plus® with a parallel and self-cutting shape, Friadent, Mannheim, Germany, *n* = 105, and Bego Semados® RI with a conical and condensing shape *n* = 28, and Bego Mini *n* = 12, Bego Implant Systems, Bremen, Germany; total *n* = 40) were inserted. Two implants were explanted at 6 (BEGO-Mini) and 34 months (Xive) after implantation, respectively. The overall implant survival rate over 189 months as determined by Kaplan–Meier Analysis was 98.7% (Figure 3).

### 3.2. Clinical and Radiological Characteristics of Dental Implants

Clinical and radiological evaluation was performed for 155 implants. Two implants were lost due to explanation. The following parameters were examined: plaque index, probing depth, bleeding on probing, implant mobility, and keratinized gingiva.

Inspection and probing revealed no plaque on 67 (43.8%) implants, grade 1 plaque according to the Mombelli index [31] on 48 (31.4%) implants, grade 2 plaque on 32 (20.9%) implants, and grade 3 plaque on 6 (3.9%) implants.

In total, 128 implants had maximum probing depths of 1.0–4.0 mm, which are considered to be physiologically normal. Probing depths were ≥ 4 mm for 24 implants. At the follow-up examinations, most (*n* = 93) implants did not bleed on probing. None of the 153 dental implants examined showed mobility, as measured manually. Periotest® values for 122 implants ranged from −7 to 0, indicating good osseointegration. Twenty-five implants had scores of 1–9, indicating the requirement for clinical reevaluation. One implant had a score of 13, which represents insufficient osseointegration. Keratinized gingiva was observed around the crowns of most (*n* = 137) implants.

Bone loss was determined radiologically for each implant by comparing bone levels on postoperative and follow-up panoramic X-rays. Bone loss of 0–0.5 mm was recorded for 33 implants, and loss of 0.5–3.5 mm was recorded for 103 implants. Bone loss > 3.5 mm was observed around 17 implants.* No correlation was found between implant systems used, the type of graft, and age or sex of patients in Fischer's exact test, and bilateral correlation testing of all parameters resulted in a p-value <= 0.05.*

### 3.3. Implant Success according to the Buser Criteria

According to Buser's success criteria, five implants in our sample failed due to explantation (*n* = 2), radiolucency (*n* = 2), and dysesthesia (*n* = 1). Thus, the implant success rate according to these criteria was 96.8%.

**Figure 4:**
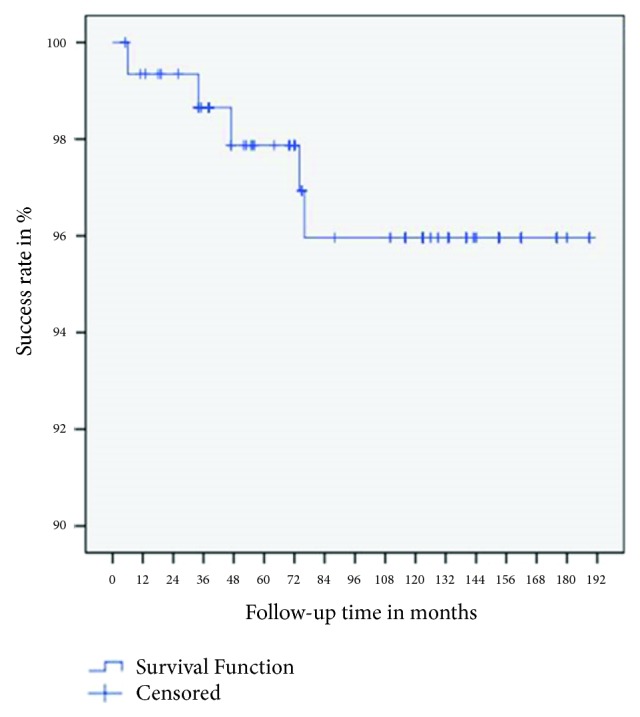
Cumulative success rate according to the Buser criteria.

The Kaplan–Meier curve for these data (Figure 4) shows that five of 155 implants failed during the 189-month (15.75-year) observation period, resulting in a cumulative success rate of 96%.

### 3.4. Implant Success according to the Albrektsson Criteria

One or more parameters (explantation, radiolucency, dysesthesia, vertical bone loss, and infection) led to the failure of 18 implants. Thus, the implant success rate according to the Albrektsson criteria was 88.4%.

**Figure 5:**
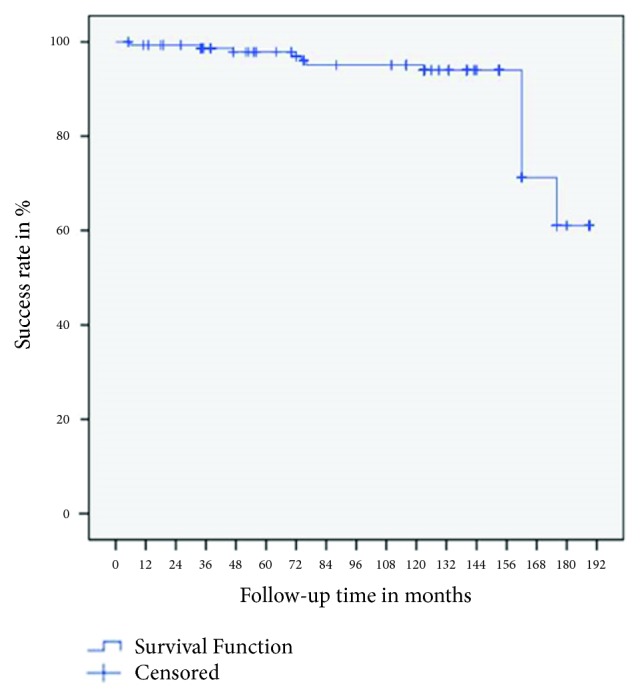
Cumulative success rate according to the Albrektsson criteria.

The Kaplan–Meier curve for these data (Figure 5) shows that 18 of 155 implants failed during the 189-month (15.75-year) observation period, resulting in a cumulative success rate of 61%.

## 4. Discussion

This study considered patients who had received dental implants due to dental aplasia, regardless of whether the condition was hypodontia, oligodontia, ectodermal dysplasia, or cleft lip or palate. Limitations of this study were related to the numbers of patients (*n* = 37) and dental implants (*n* = 155) included. This relatively small sample is not representative of a larger population. Due to the rareness of dental aplasia, smaller numbers of patients and implants were included in previous studies [27, 33, 34]. The sex distribution in this study was 43.2% male and 56.8% female patients (ratio, 1:1.3). Similar distributions have been reported in the literature [8, 9]. Patient age at the time of implantation in this study ranged from 17 to 44 years (mean, 20 years), and 89.2% (*n* = 33) of patients were aged 17–23 years. Other studies have included similarly young patient groups [35, 36]. A possible explanation for the predominance of young patients is that early implant treatment planning commences at the age of 17–21 years, when cranial growth is complete. The most frequently absent teeth replaced with dental implants in this study were the maxillary lateral incisors and maxillary and mandibular second premolars. This prevalence distribution is comparable to results from the literature [7, 9]. The implant loss rate in this study was 1.3% (*n* = 2), and the* in situ* implant survival rate was 98.7% (*n* = 153/155). One implant was lost after 34 months due to osseointegration failure, and another was lost after 6 months due to peri-implantitis. Notably, the latter was a mini-implant with a diameter of 2.9 mm and length of 11.5 mm. Becelli et al reported a survival rate of 96.6% for 60 implants in 8 oligodontic patients [34]. In a review and meta-analysis of 19 articles on this topic, survival rates ranged from 76.6% to 100%, and the overall survival rate was 95.3% [37]. Comparable results were recorded in the present study.

### 4.1. Implant Success Criteria

The survival of dental implants is not necessarily equivalent to their success. The assessment of implant success in addition to survival is very important in the evaluation of treatment outcomes. Many similar studies of dental implants in patients with dental aplasia did not involve the use of implant success criteria [37]. In studies assessing implant success, self-defined criteria or the sole criterion of the marginal bone level had been applied [38–40]. Therefore, comparison of implant success rates between this study and previous studies is not meaningful. In this study, implant success was evaluated using the criteria of Buser et al. [30] and Albrektsson et al. [29]. Two sets of criteria were used to enable the consideration of differences in implant success in an identical patient sample resulting from the use of different measures. Depending on the criteria used, the implant success rate in the same patient sample may vary. According to the Buser criteria, five implants in this study were considered to have failed (two due to radiolucency and one due to paresthesia). According to the Albrektsson criteria, an additional 13 implants from the same sample failed (total,* n*= 18). Unlike the Buser criteria, the Albrektsson criteria include vertical bone loss and the presence of infection (peri-implantitis). As a result, the peri-implant hard and soft tissues were evaluated more stringently, which explains the lower success rate. However, neither of these criteria includes subjective assessment of dental implants. Only the success criteria of Jahn and d'Hoedt [41] consider patient satisfaction. Buch et al. criticized the use of only hard- and soft-tissue evaluations for the assessment of implant success and recommended additional subjective assessment of patient satisfaction [42]. About 30 years ago, researchers used primarily measurable clinical parameters to detect disease-related impairments and evaluate therapeutic success; today, patients' perceived satisfaction has become focal [43]. For this reason, patient satisfaction should be taken into consideration in the future establishment of success criteria. The Buser and Albrektsson criteria also neglect the assessment of prosthetic outcome, which should be considered in future development of success criteria. A new implant success assessment tool could also employ score calculation in which criteria (clinical and radiological parameters, prosthetic outcome, and patient satisfaction) are differentially weighted statistically. The classification of implant success should be graded (e.g., very good, good, medium, and bad), so that a less successful implant does not necessarily mean complete failure.

## 5. Conclusion

In this retrospective study, 155 implants were inserted in patients with dental aplasia (risk group) and examined during a median observation period of 10.25 years. The survival rate (98.7%) was comparable to those of other studies conducted with normal cohorts. Patient satisfaction parameters are planned to be acquired, addressed, and discussed in a future manuscript. In this study, two sets of criteria were used to measure implant success. The implant success rate was higher according to the Buser criteria (96.8%) than according to the Albrektsson criteria (88.4%). The main reason for the lower Albrektsson implant success rate is the assessment of marginal bone loss. Further development of a complex implant success scoring system might be useful for standardized follow-up evaluation of dental implants.

## Figures and Tables

**Table 1:** Summarize the clinical and radiological parameters with the selected scoring system.

Clinical/Radiological Parameters	Scoring system
Plaque index	the modified Mombelli plaque index [31]: (i) Grade 0: no plaque detected by inspection and probing. (ii) Grade 1: accumulation of plaque that is visible only by probing the sulcus with a probe but not with the eye. (iii) Grade 2: visible plaque deposition. Grade 3: massive plaque deposition.

Probing depth	measured using the Click-Probe (Kerr Corporation, Orange, CA, USA) The measurement takes place in four sites around the implant - mesial, vestibular, distal and oral. The maximum measurements in mm were recorded.

Bleeding on probing	The bleeding index is determined parallel to the probing depths. If bleeding occurs during probing, this is indicated in the patient's sheet with a plus sign [+]

Mobility grade/Osseointegration	Periotest® device (Gulden, Modautal, Germany) The manufacturer specifies a scale from -08 to +50. The smaller the measured value is, the better the osseointegration is assessed: (i) Values from -08 to 00: good osseointegration of the implant (ii) Values +01 to +09: A clinical review is needed to investigate osseointegration (iii) Values from +10 to +50: Insufficient osseointegration of the implant

Vertical bone loss	the difference in alveolar bone height between panoramic X-rays taken immediately after implantation and at the follow-up examination in millimeters

## Data Availability

The data used to support the findings of this study are available from the corresponding author upon request.
